# Evaluation of LVEDP Change During High-Risk PCI With and Without Impella Support (ELVIS)—A Pilot Trial

**DOI:** 10.3390/jcm14030824

**Published:** 2025-01-27

**Authors:** Juergen Leick, Anida Gjata, Jan Pulz, Tobias Weisbarth, Kristof Richter, Tobias Krause, Louai Saad, Rashad Zayat, Philipp Kolat, Assad Haneya, Jan-Malte Sinning, Nikos Werner

**Affiliations:** 1 Heart Center Trier, Department of Cardiology, Barmherzige Brueder Hospital, Nordallee 1, 54296 Trier, Germany; a.gjata@bbtgruppe.de (A.G.); t.krause@bbtgruppe.de (T.K.); m.saad@bbtgruppe.de (L.S.); n.werner@bbtgruppe.de (N.W.); 2 Department of Cardiology, Cellitinnen Hospital St. Vinzenz Cologne, Merheimer Straße 221-223, 50733 Cologne, Germany; jan.pulz@cellitinnen.de (J.P.); tobias.weisbarth@cellitinnen.de (T.W.); kristof.richter@cellitinnen.de (K.R.); jan-malte.sinning@cellitinnen.de (J.-M.S.); 3 Heart Center Trier, Department of Cardiothoracic Surgery, Barmherzige Brueder Hospital, Nordallee 1, 54296 Trier, Germany; r.zayat@bbtgruppe.de (R.Z.); p.kolat@bbtgruppe.de (P.K.); a.haneya@bbtgruppe.de (A.H.)

**Keywords:** high-risk percutaneous coronary intervention, left ventricular end-diastolic pressure, percutaneous mechanical circulatory support

## Abstract

**Background:** The decision-making process to use percutaneous mechanical circulatory support in the context of elective high-risk percutaneous coronary intervention (HRPCI) is complex and evolving. The aim of this study is to evaluate the left ventricular end-diastolic pressure (LVEDP) as a parameter to identify patients that may benefit from a protected HRCPI (pHRPCI) procedure. **Methods:** Overall, 62 patients (pHRPCI *n* = 31 vs. non-pHRPCI *n* = 31) with a complex coronary artery disease and a left ventricular ejection fraction (LVEF) ≤35% were included. The primary endpoint was defined as a change in LVEDP and its correlation with laboratory measurements. The secondary safety endpoint was a composite of the incidence of major in-hospital adverse cardiac and cerebrovascular events (MACCE). **Results:** Baseline characteristics were similar, including age (pHRPCI 72.8 ± 8.8 vs. non-pHRPCI 75.0 ± 10.4; *p* = 0.408), male (pHRPCI 83.9% vs. non-pHRPCI 96.8; *p* = 0.195), pre-PCI Syntax Score (pHRPCI 33.9 ± 13.1 vs. non-pHRPCI 35.4 ± 12; *p* = 0.643), post-PCI Syntax Score (pHRPCI 7.4 ± 6.2 vs. non-pHRPCI 9.6 ± 8.4; *p* = 0.239) and baseline LVEDP between the groups (pHRPCI 18.5 ± 10.5 mmHg vs. non-pHRPCI 15.7 ± 8.1 mmHg; *p* = 0.237). There was a trend to a lower LVEF in the pHRPCI group (26.4 ± 6.7% vs. 29.4 ± 5%; *p* = 0.051). The primary endpoint analysis revealed a significant change in LVEDP (pHRPCI −4.7 ± 9 mmHg vs. non-pHRPCI +3.1 ± 7.5 mmHg; *p* < 0.001) that did not correlate with changes in creatinine (*p* = 0.285), NT-proBNP (*p* = 0.383) or troponin (*p* = 0.639) concentrations within 24 h. Overall, low rates of in-hospital (pHRPCI 6.5% vs. non-pHRPCI 3.2%; *p* = 0.999) and 90-days (pHRPCI 12.9% vs. non-pHRPCI 12.9%; *p* = 0.999) MACCE were observed in both groups. **Conclusions:** Protected HRPCI leads to a significant reduction in LVEDP without influencing biomarkers of myocardial damage. There was no difference in MACCE rates between the groups.

## 1. Introduction

Percutaneous coronary intervention (PCI) of patients with impaired left ventricular ejection fraction (LVEF) and complex or severe coronary artery disease (CAD), in whom surgical treatment of the coronary arteries cannot be performed due to anatomical reasons, represents a challenge for interventional cardiologists and requires optimal hemodynamic monitoring. In the context of percutaneous high-risk coronary intervention (HRPCI) in this patient population, the use of percutaneous mechanical circulatory support (pMCS) may be considered to improve patient safety [1,2].

The indication for pMCS in the context of HRPCI is still a matter of debate. Currently, the indication is based on the criteria of the PROTECT II trial, results from registry studies, expert consensus, and personal experience [1,2,3,4,5,6,7]. Although the use of pMCS is well established in HRPCI, there is no clear guideline recommendation for indication due to limited published data [8]. The main indication for pMCS is currently an expected deterioration of hemodynamic during a complex HRPCI procedure. However, clear parameters are not defined, which may predict the risk of periprocedural hemodynamic deterioration in a HRPCI patient population [9]. A depressed LVEF in combination with comorbidities and high-risk features of the coronary arteries may not be sufficient for decision-making. Moreover, an easy-to-evaluate parameter that can be used by the interventional cardiologist for decision-making is currently lacking. Left ventricular end-diastolic pressure (LVEDP) may play a decisive role as a parameter in this context. An elevated LVEDP reflects increasing left ventricular wall tension and can serve as an indicator of potential hemodynamic instability, myocardial dysfunction, or cardiogenic shock. Increased LVEDP is associated with progressive myocardial ischemia, acute kidney injury/acute renal failure, and a reduction in mean arterial pressure (MAP) and cardiac output (CO). To date, there are no prospective studies investigating the changes in LVEDP in HRPCI. In addition, the data from retrospective studies are contradictory [9,10]. In the present pilot study, systematic analyses of pre-, peri-, and post-interventional LVEDP values and its changes were performed in combination with evaluation of cardiac necrosis markers and markers of renal function and clinical outcomes.

## 2. Methods

### 2.1. Patient Selection

**Figure 1:**
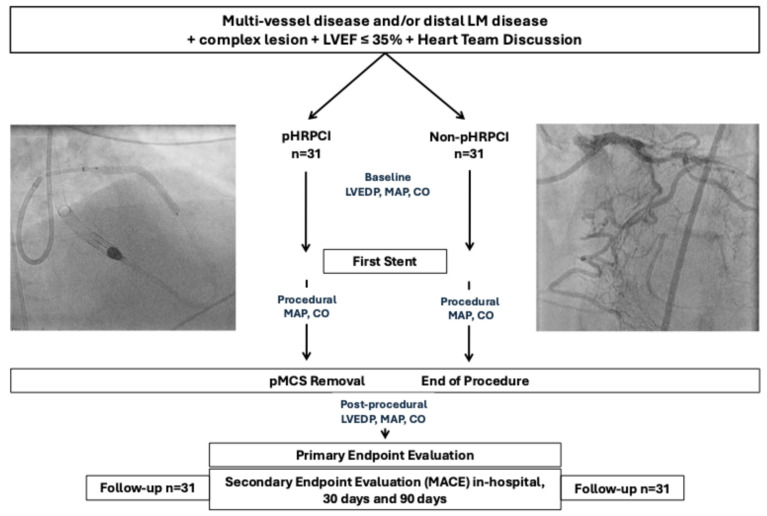
Study design. Abbreviations: cardiac output (CO); left ventricular end-diastolic pressure (LVEDP); left ventricular ejection fraction (LVEF); mean arterial blood pressure (MAP); non-protected high risk percutaneous coronary intervention (non-pHRPCI); protected high risk percutaneous coronary intervention (pHRPCI). pMCS = percutaneous mechanical circulatory support.

This pilot study was designed for hypothesis-generating. The study was approved by the ethics committee of the Rhineland-Palatinate Chamber of Physicians (Number 2021-15611). Patient recruitment occurred in the Heart Center Trier and the St. Vinzenz Hospital Cologne between June 2021 and June 2024. All patients with an LVEF ≤35% and an elective HRPCI for whom the decision for interventional revascularization and a pMCS had been made jointly by the interdisciplinary heart team, were included (Figure 1). Due to a lack of standardized criteria and studies, the patient-relevant criteria (age, frailty, quality of life), complexity of CAD and other anatomical conditions, were included. The selection of patients for protected-HRPCI (pHRPCI) was largely based on the recently published recommendations of a European expert group [4].

The definition of complex coronary intervention was adapted from the criteria published by Riley et al. [3]. All patients had at least one of the following angiographic criteria: coronary three-vessel disease and planned intervention of at least two coronary vessels with at least three points in the complexity score or a distal left main (LM) stenosis with a planned ≥2 stent strategy and at least one point in the complexity score. The complexity score was calculated as follows: 1 point for a long-segment stenosis (>24 mm); 2 points for a bifurcation stenosis (without LM involvement) with a planned ≥2 stent strategy; 3 points for a calcified stenosis with indication for rotablation/orbital atherectomy or intravascular lithotripsy; and 3 points for a PCI in a last remaining coronary vessel.

The exclusion criteria were defined as follows: significant aortic valve stenosis, mechanical aortic valve replacement, ventricular septal defect, ventricular thrombus, severe peripheral artery disease (relative contraindication), severe renal insufficiency (stage 4; Kidney Disease Outcomes Quality Initiative [KDOQI] classification) defined as estimated glomerular filtration rate (eGFR) <29 mL/min/1.73 m^2^, pregnancy, and lack of informed consent or patient incapable of giving consent. The data for the 30-day and 90-day follow-ups were taken from the clinic’s internal quality control for HRPCI.

### 2.2. PMCS Insertion and Removal

All patients in the pHRPCI group had the Impella^®^ CP Smart Assist (Abiomed/Johnson & Johnson Med Tech, Aachen, Germany) inserted prior to PCI via the left or right femoral artery according to the manufacturer’s recommendation. In all patients, 1 or 2 Proglide™ (Abbott Medical GmbH, Wetzlar, Germany) closure systems were placed in advance for post-procedural access site closure. Once the Impella was positioned, it was activated via the Automatic Impella Controller and the automatic Impella algorithms (Impella CP with Smart Assist) was started. After the procedure, if the patient was hemodynamically stable, the Impella flow was gradually reduced and the Impella was removed in the catheterization laboratory. Access site closure was performed using the Proglide devices and an angiographic control by an ipsilateral microinjection or via the radial artery, which was strongly recommended to rule out acute vascular complications.

### 2.3. Coronary Intervention

The intervention was performed by a cardiologist experienced in coronary intervention with the additional qualification, “interventional cardiology”, in accordance with the curriculum of the German Society of Cardiology. The PCI strategy, as well as further antiplatelet therapy, was at the discretion of the interventional cardiologist.

### 2.4. Hemodynamic Measurements

Hemodynamic measurements were captured in all patients. The MAP, CO, mean pulmonary artery pressure (mPAP), pulmonary capillary wedge pressure (PCWP), and LVEDP were measured 3× at the beginning of the procedure and 3× at the end of the procedure/after removal of the pMCS (Figure 1). Additional MAP, CO, mPAP, and PCWP measurements were performed after the first drug eluting stent (DES) implantation or drug-coated balloon application. The standardized measurement of LVEDP was performed using a 5 or 6 French pigtail catheter and CO was measured by the estimated oxygen uptake Fick (eFick) method.

### 2.5. Laboratory Measurements

The blood samples for the laboratory analysis were taken at time of access site puncture (baseline) and 6 h, 12 h, and 24 h post-puncture. The following values were analyzed: high-sensitive cardiac troponin T (hs-cTnT), creatinine kinase (CK), creatinine kinase isoenzyme–MB (CK–MB), N-terminal pro brain natriuretic peptide (NT-proBNP), creatinine, and eGFR.

### 2.6. Primary and Secondary Endpoint Definition

The primary endpoint was defined as a change in LVEDP (baseline LVEDP–post-PCI LVEDP) and its correlation with markers of the renal function, hs-cTnT and NT-proBNP.

The secondary safety endpoint was a composite of in-hospital cardiac death, acute myocardial infarction (AMI) and cerebrovascular events (MACCE). For the definition of peri-interventional AMI (type 4a), the criteria of the Fourth Universal Definition of Myocardial Infarction were applied [11].

### 2.7. Statistical Analysis

The data were processed, and the mean values and standard deviations of the metric variables were presented as part of the descriptive statistics. The absolute and relative frequencies were determined for the non-metric variables. The data were checked for the presence of bias, which could not be confirmed, so a correction using a propensity score was not required. For the analysis, *t*-tests were used for dependent and independent variables. Analyses of variance (ANOVA) were used for comparison of four measurement points with consideration of main effect and interaction and, if necessary, Greenhouse–Geisser correction.

Prerequisites for application were checked for each method used. These included the distribution assumptions depending on the sample size, homogeneity of variances, sphericity and multicollinearity. Where necessary, non-parametric tests were performed. Depending on the respective violated condition, the Wilcoxon rank sum test, Welch test, Friedman test and resampling in the sense of Bootstrapping, were considered. A cut-off of 19 mmHg was selected to test the influence of using the pMCS on the laboratory values as a function of LVEDP. This value was determined by an iterative converging procedure based on the group differences in a data-driven manner. Unless otherwise stated, two-sided tests were performed, a significance level corresponding to α = 0.05 was tested. The evaluation was carried out with R 4.3.2 (R Core Team, 2023).

## 3. Results

### 3.1. Baseline and Procedural Characteristics

A total of 62 consecutive patients were included in the study. Of these, 31 patients were assigned to the pHRPCI and 31 to the non-pHRPCI group, respectively. Baseline clinical characteristics and procedural characteristics are shown in Table 1. Most of the patients in each group were male, with a mean age of 72.8 ± 8.8 and 75.0 ± 10.4 years old in the pHRPCI group and the non-pHRPCI group, respectively (*p* = 0.408). There was a trend to a lower baseline LVEF in the pHRPCI group (26.4 ± 6.7% vs. 29.4 ± 5%; *p* = 0.051). Overall, no significant differences were observed in any baseline co-morbidities or CAD presentation.

In contrast, procedure duration (147 ± 39.8 min vs 107.8 ± 30.5 min; *p* < 0.001) and fluoroscopy time (49.1 ± 15.9 min vs. 37.6 ± 15.5 min; *p* = 0.006) were significantly longer in the pHRPCI group compared to the non-pHRPCI group. No significant differences in the need for intraprocedural vasopressors was observed between the pHRPCI and non-pHRPCI groups (16.1% vs. 25.8%, *p* = 0.533). Additionally, unexpected blood pressure drops, defined as a ≥20 mmHg systolic pressure drop, were similar between groups (pHRPCI 0.81 ± 1.8 vs. non-pHRPCI 0.4 ± 0.5; *p* = 0.256) and no correlation between drops in blood pressure and the baseline LVEDP (*p* = 0.256) were observed.

Overall, the peri-interventional complication rate was low. One patient in the pHRPCI group developed significant bleeding at the access site in the common femoral artery requiring blood transfusion (*p* = 0.999). Four patients in the pHRPCI group had bleeding at the access site without the need for further measures (*p* = 0.113). Furthermore, no relevant peripheral ischemia or acute coronary complication occurred during the PCI procedure in either patient group.

### 3.2. Right Heart Catheterization and Laboratory Measurements

The data related to right heart catheterization are reported in Table 2. We observed a higher CO in patients from the pHRPCI group compared to the non-pHPCI group during the procedure (pHRPCI 4.7 ± 1 L/min vs. HRPCI 4.0 ± 1.1 L/min; *p* = 0.014). In addition, we observed an increase in PCWP in the non-pHRPCI group (baseline 13.7 ± 7.7 mmHg vs. post-PCI 17.7 ± 9.3 mmHg) and a trend towards change in PCWP between groups (pHRPCI 0.07 ± 9.6 mmHg vs. non-pHRPCI 4.33 ± 7.8 mmHg; *p* = 0.061).

The data related to laboratory measurements are reported in Table 3. The NT-proBNP, hs-cTnT and creatinine kinase isoenzyme–MB concentrations at baseline, 6 h, 12 h, and 24 h post-procedure were not statistically different between groups. However, we did observe a higher concentration of creatinine (1.5 ± 0.5 mg/dL vs. 1.2 ± 0.4 mg/dL; *p* = 0.043) and a lower eGFR (53.5 ± 19.8 mL/min/1.73 m^2^ vs. 65.9 ± 21.5 mL/min/1.73 m^2^; *p* = 0.022) after 24 h in the pHRPCI group. This was also evident with delta creatinine (pHRPCI 0.3 ± 0.3 mg/dL vs. non-pHRPCI 0.2 ± 0.2 mg/dL; *p* = 0.039) and delta GFR (pHRPCI −16.6 ± 12.9 mL/min/1.73 m^2^ vs. non-pHRPCI −10.4 ± 9.3 mL/min/1.73 m^2^; *p* = 0.033).

No significant differences were observed in NT-proBNP levels, creatinine kinase–MB, and hs-cTnT levels after 24 h between the pHRPCI and non-pHRPCI groups. Although there was a significant increase in hs-cTnT within 24 h in the pHRPCI group, there was no difference in delta hs-cTnT (253.3 ± 532.9 pg/mL vs. 136.2 ± 135.1 pg/mL; *p* = 0.303). There was a trend towards a lower delta NT-proBNP in the pHRPCI group within the first 12 h (1615 ± 2491 pg/mL vs. 3127 ± 10.190 pg/mL; *p* = 0.48).

### 3.3. Primary Endpoint Analysis

**Figure 2:**
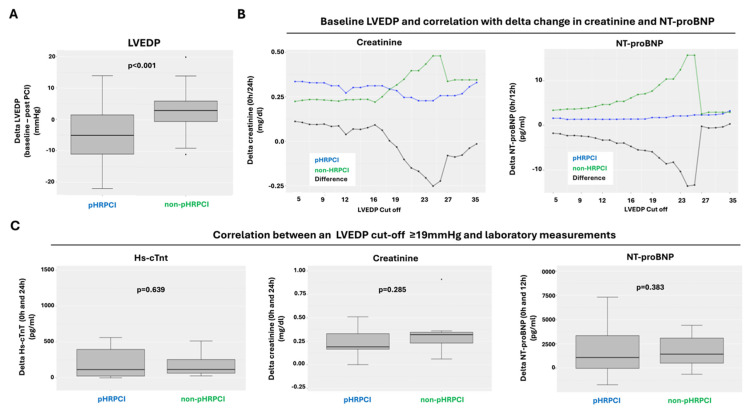
Primary endpoint analyses. (**A**) Primary outcome showing delta LVEDP between the pHRPCI and non-pHRPCI group. Changes in (**B**) creatinine (0 h and 24 h) and NT-proBNP (0 h and 12 h) from baseline LVEDP. (**C**) Changes in hs-cTnT (0 h and 24 h), creatinine (0 h and 24 h), and NT-proBNP (0 h and 12 h) from baseline LVEDP ≥19 mmHg. Abbreviations: hs-cTnT: high-sensitive cardiac troponin T; LVEDP: left ventricular end-diastolic pressure; NT-proBNP: N-terminal pro brain natriuretic peptide; non-pHRPCI: non-protected high risk percutaneous coronary intervention; pHRPCI: protected high risk percutaneous coronary intervention.

Analysis of the primary endpoint revealed a significant change in LVEDP after the procedure between pHRPCI and non-pHRPCI patients (−4.7 ± 9 mmHg vs. 3.1 ± 7.5 mmHg; *p* < 0.001; Figure 2A). After correlation with the laboratory values, we observed nearly constant NT-proBNP values and lower creatinine values in the pHRPCI group, depending on the baseline LVEDP (Figure 2B). A cut-off was set at a LVEDP of 19 mmHg based on this analysis. No correlation was observed between an LVEDP of ≥19 mmHg and delta hs-cTnT (*p* = 0.639), delta creatinine (*p* = 0.285), NT-proBNP (*p* = 0.383), as well as eGFR (*p* = 0.401) (Figure 2C).

### 3.4. Secondary Endpoint Analysis

Analysis of safety endpoints demonstrated low rates of in-hospital MACCE in both groups, with 6.5% MACCE reported in pHRPCI (*n* = 2) patients and 3.2% reported in non-pHRPCI (*n* = 1) patients (*p* = 0.999). In the pHRPCI group, two patients fulfilled the criteria of an AMI type 4a, while intra-hospital cardiac death was reported in one patient from the non-pHRPCI group. During the 90-day follow-up there were no differences in all-cause mortality (pHRPCI 6.5% vs. non-pHRPCI 12.9%; *p* = 0.671) and the rate of cardiac death (pHRPCI 6.5% vs. 9.7%; *p* = 0.999) between the groups. AMI occurred within 90 days in 6.5% of the cases in each group (*p* = 0.999). In the non-pHRPCI group, one patient suffered a stroke. Overall, after 90 days, the MACCE rate was 12.9% in both groups (*p* = 0.999).

## 4. Discussion

To our knowledge, this is the first prospective study investigating the clinical relevance of LVEDP during HRPCI to determine procedural efficacy and help identify patients at risk of peri- and/or post-interventional hemodynamic deterioration. The decision-making process to use pMCS in the context of elective HRPCI is highly debated. European expert groups and guidelines recommend that the use of pMCS in HRPCI should at least be considered in patients with depressed LVEF and features of complex high-risk coronary artery stenoses [4,12]. The American College of Cardiology, American Heart Association, and Society for Cardiovascular Angiography and Interventions gives a class IIb recommendation [13], but a clear recommendation of the European Society of Cardiology guideline for Myocardial Revascularization is currently lacking [8]. Since there are no clear recommendations to identify which patients may be indicated for pMCS in the context of elective HRPCI, the aim of the current study was to examine whether LVEDP at baseline or its change during HRPCI, is helpful as a simple, cost-effective, and point-of-care parameter in the decision-making process. As such, we observed a significant reduction in LVEDP in the pHRPCI group after PCI and removal of the pMCS. Moreover, there was a tendency for patients with a baseline LVEDP of ≥19 mmHg to benefit from a reduction in LVEDP. Both groups reported low intrahospital complication rates. Collectively, this pilot study is hypothesis-generating and could form the basis for a larger prospective study to evaluate potential LVEDP cut-offs for patient selection for pHRPCI.

### 4.1. LVEDP Change in the Protected HRPCI and Non-Protected HRPCI Group

Previous studies have shown that there is an unfavorable correlation between LVEDP and poor long-term outcomes in patients with acute ST-elevation myocardial infarction [14,15]. Moreover, increased LVEDP in patients with AMI has been shown to be an independent predictor of mortality or reinfarction [14]. However, the reported specificity (72.5%) and sensitivity (68.3%) are low [15]. As such, a study of 49,600 patients undergoing PCI, identified LVEDP as an independent predictor of adverse outcomes, with a LVEDP of ≥26 mmHg leading to a 10.4% higher in-hospital mortality rate in these patients [16]. However, this study combined elective patients with emergency patients (52%) and the proportion of patients with an LVEF of ≤35% was only 11% with pMCS used in less than 2% of the cases. None of the studies addressed the clinical problem of HRPCI and evaluating LVEDP as a marker for left ventricular unloading. Here, we observed that the pHRPCI patients in our study had a significant reduction in LVEDP during the procedure and this remained constant after removal of the pMCS. The pHRPCI group also had a higher CO value during PCI. This was considered to be an effect of the Impella by unloading the left ventricle and thereby decreasing left ventricular work and increasing the cardiac output. In comparison, the PCWP values and change in the non-pHRPCI group tended to increase during the intervention. This suggests hemodynamic deterioration in the non-pHRPCI group. However, the PCPW remained largely constant during the PCI in the pHRPCI group. In this context, a study including LVEDP and PCWP data from more than 3900 patients reports that the PCWP measurements slightly underestimated LVEDP values by 2.9 mmHg [17]. This could also be observed in a study that investigated the correlation of LVEDP and PCWP during exercise [18]. Despite a good correlation, these two measurements may occasionally differ, so this could explain the differences in our cohort.

### 4.2. Correlation of LVEDP with Markers of Myocardial Damage and Renal Function

In the current study, we observed a trend towards a lower creatinine increase and a more stable NT-proBNP concentration (Figure 2B) in the pHRPCI group. Based on this analysis, a baseline cut-off of 19 mmHg was chosen. However, there was no statistically significant correlation between the baseline LVEDP and the changes in the laboratory values (Figure 2C).

Recently, an inverse correlation between LVEDP and contrast-induced nephropathy in patients undergoing PCI with a cut-off of 14.5 mmHg has been reported [19]. However, we were unable to identify this association in our study (Figure 2B). In contrast, we observed that there was a significant increase in creatinine within 24 h in the pHRPCI group. We attributed this to the tendency of the pHRPCI patients to have increased needs for higher contrast fluid administration during the angiography of the access site recommended in the study protocol prior to insertion and removal of the pMCS. This was performed in the pHRPCI group only. Compared to other pMCS studies [2,10], the amount of contrast agent used (ranging from 267 ± 142 to 274 ± 105 mL) did not differ with our cohort (260 ± 98 mL). Interestingly, in the Danish–German Cardiogenic Shock (DanGer Shock) Trial, the proportion of patients with acute renal failure was also higher in the pMCS (41.9%) group than in the control group (26.7%) [20]; this was likely the result of mechanical hemolysis caused by the microaxial flow pump of the Impella. This can lead to an increase in plasma free hemoglobin and can trigger nephropathy, which can result in acute renal failure [20]. However, our pMCS duration was limited to the time of PCI in elective treated patients and therefore cannot be directly compared to the DanGer Shock Trial.

In the non-pHRPCI group, we observed a trend towards higher NT-proBNP values depending on the baseline LVEDP within the first 12 h (Figure 2B), which is in line with previously published literature [21]. In patients with pHRPCI, the NT-proBNP difference in relation to baseline LVEDP remained largely constant, which we attributed to the significant reduction in LVEDP in this group (Figure 2A). This hypothesis is supported by the fact that other factors that can lead to an increased NT-proBNP, such as the sudden drop in blood pressure or the need for vasopressor administration, occurred equally in both groups. Additionally, we examined whether occurrence of sudden systolic blood pressure drops during HRPCI could be predicted by baseline LVEDP. A significant correlation was ruled out, which is also comparable with a previous retrospective study that found no association between LVEDP <15 mmHg and ≥15 mmHg and the likelihood of peri-interventional blood pressure drop in patients with pMCS [10]. However, these results must be compared with caution given the different cut-off values that were analyzed. Finally, we were unable to demonstrate a correlation between baseline LVEDP and hs-cTnT concentration. We attribute this to the high degree of complete revascularization in both groups, which was reflected in the post-PCI Syntax Scores (pHRPCI 7.4% vs. non-pHRPCI 9.6%; *p* = 0.239).

### 4.3. Secondary Endpoint

Overall, both groups had low in-hospital MACCE rates (pHRPCI 6.5% vs. non-pHRPCI 3.2%), which were significantly lower than in the Protect II (10.2%) study and comparable to the Protect III study (5%) [1,2]. During the 90-day follow-up, the MACCE rate was 12.9% in both groups. This is comparable to the data from the PROTECT III study (15%) and also lower than in the Protect II study (22%) [1]. Standardized procedures of pMCS insertion and removal have been advocated in recent years with the usage of pre-closure devices, rigorous anticoagulation regimens, and angiographical control after removal, which is probably reflected by the lower MACCE rates [1]. These standardized insertion and removal methodologies of the pMCS could also explain the longer procedure duration and fluoroscopy time due to the non-pHRPCI patient group. Similarly, the procedure duration in the Protect II study does not differ from the current study [2]. There are no comparable 90-day data on the non-pHRPCI group in the literature. The lack of difference in the 90-day MACCE rate between the groups could be due to the high degree of revascularization in both groups.

## 5. Limitations

Due to the limited size of the patient cohort, only trends in the delta LVEDP and its correlation with laboratory values can be stated. The secondary endpoint was chosen to compare the data with the Protect II and Protect III studies [1,2]. The cut-off value of ≥19 mmHg was determined iteratively as a heuristic parameter. The iterative calculations performed, and their visualization (Figure 2B) indicate the presence of a cut-off value. Due to this statistical approach, no sensitivity can be given for the cut-off value of ≥19 mmHg. However, as this is a hypothesis-generating pilot study, this was considered in the planning. Although the overall cohort was balanced in the statistical analysis, a bias cannot be ruled out, particularly since the patients were selected by an interdisciplinary heart team decision and not by randomization. This is particularly evident in the baseline LVEF. There was a trend towards a lower LVEF in the pHRPCI group. Additionally, since next-day discharge occurred in most patients, 48 h post-procedure creatinine is missing as well as data on acute kidney injury in this cohort. Moreover, analysis of the laboratory values was limited to 24 h of collected laboratory measurements.

## 6. Conclusions

Protected HRPCI leads to a significant reduction in LVEDP, which is not observed in non-pHRPCI patients. Although LVEDP did not correlate significantly with creatinine, NT-proBNP or Hs-cTnT levels, we observed a trend based on a cut-off of ≥19 mmHg, suggesting that these non-pHRPCI patients may benefit from pHRPCI [22]. However, there was no difference between the groups in the 90-day MACCE rate. As this pilot study was designed to be hypothesis-generating, a further randomized study should be conducted to identify an LVEDP cut-off value.

## Figures and Tables

**Table 1:** Patient and Procedural Characteristics.

Characteristic	pHRPCI (*n* = 31)	Non-pHRPCI (*n* = 31)	*p*-Value
**Baseline characteristics**			
Male, *n* (%)	26 (83.9)	30 (96.8)	0.195
Age, years mean ± stdv	72.8 ± 8.8	75.0 ± 10.4	0.408
BMI, kg/m^2^ mean ± stdv	26.3 ± 3.5	26.7 ± 5.1	0.73
Hypertension, *n* (%)	26 (83.9)	29 (93.5)	0.425
Nicotine abuse, active, *n* (%)	4 (12.9)	9 (29.0)	0.211
Former nicotine abuse, *n* (%)	11 (35.5)	12 (38.7)	0.999
Diabetes mellitus, *n* (%)	13 (41.9)	11 (35.5)	0.794
Hyperlipoproteinemia, *n* (%)	26 (83.9)	27 (87.1)	0.999
Family history of CAD, *n* (%)	10 (32.3)	11 (35.5)	0.999
Chronic kidney disease, *n* (%)	15 (48.4)	14 (45.2)	0.999
**CAD and clinical presentation**			
3-CAD with PCI *, *n* (%)	28 (90.3)	24 (77.4)	0.301
LM PCI included, *n* (%)	15 (48.4))	16 (51.6)	0.999
Syntax Score pre-PCI mean ± stdv	33.9 ± 13.1	35.4 ± 12	0.643
Syntax Score post-PCI mean ± stdv	7.4 ± 6.2	9.6 ± 8.4	0.239
Previous CABG, *n* (%)	1 (3.2)	4 (12.9)	0.354
Log Euro Score mean ± stdv	9.5 ± 12.9	11.6 ± 12.6	0.509
LVEF at baseline, mean ± stdv	26.4 ± 6.7	29.4 ± 5.0	0.051
LVEF at discharge, mean ± stdv	32.1 ± 7.4	32.5 ± 7.0	0.853
Left ventricular volume at discharge, ml mean ± stdv	179 ± 60.9	161.6 ± 49.0	0.233
**Procedural Characteristics**			
PCI of two coronary arteries, *n* (%)	18 (58.1)	17 (54.8)	0.999
PCI of three coronary arteries, *n* (%)	12 (38.7)	12 (38.7)	0.999
Cumulative stent length, mm, mean ± stdv	117.1 ± 42.2	104.8 ± 37	0.229
Contrast volume, mL, mean ± stdv	260.1 ± 89.4	231.9 ± 70.2	0.172
Rotational atherectomy, *n* (%)	4.0 (12.9)	4.0 (12.9)	0.999
Orbital atherectomy, *n* (%)	1.0 (3.2)	1 (3.2)	0.999
Intravascular lithotripsy, *n* (%)	4.0 (12.9)	6.0 (19.4)	0.731
Procedure time, min, mean ± stdv	147.3 ± 39.8	107.8 ± 30.5	<0.001
Fluoroscopy time, min, mean ± stdv	49.1 ± 15.9	37.6 ± 15.5	0.006

Abbreviations: BMI: body mass index; CAD: coronary artery disease; CABG: coronary artery bypass graft surgery; HRPCI: high-risk percutaneous coronary intervention; LVEF: left ventricular ejection fraction; PCI: percutaneous coronary intervention; pHRPCI: protected high-risk percutaneous coronary intervention. * ≥2 vessels and ≥3 points in complexity score.

**Table 2:** Hemodynamics.

Variable, Mean ± Stdv	pHRPCI (*n* = 31)	Non-pHRPCI (*n* = 31)	*p*-Value
**LVEDP, mmHg**			
Baseline, mmHg	18.5 ± 10.5	15.7 ± 8.1	0.237
Post-procedure, mmHg	13.8 ± 8.9	18.8 ± 9.5	**0.036**
Delta change, mmHg	−4.7 ± 9	3.1 ± 7.5	**<0.001**
**Mean arterial pressure, mmHg**			
Baseline, mmHg	86.2 ± 15	79.4 ± 16.5	0.096
After first DES, mmHg	86.8 ± 17.4	85.8 ± 13.1	0.799
Post-procedure, mmHg	89.3 ± 16.7	88.2 ± 14.1	0.787
≥20 mmHg blood pressure drop event	0.81 ± 1.8	0.42 ± 0.50	0.256
**Right heart catheterization, mmHg**			
mPAP baseline, mmHg	26.8 ± 12	22.2 ± 8	0.2
mPAP after first DES, mmHg	28.5 ± 10.8	25.5 ± 10.7	0.272
mPAP post-procedure, mmHg	26.2 ± 11	25.2 ± 10.0	0.787
PCWP baseline, mmHg	17.0 ± 9.8	13.7 ± 7.7	0.151
PCWP after first DES, mmHg	17.8 ± 9.1	16.2 ± 8.6	0.482
PCWP post-procedure, mmHg	17.1 ± 8.4	17.7 ± 9.3	0.769
Cardiac output baseline, L/min	4.4 ± 1.0	4.6 ± 2.3	0.688
Cardiac output after first DES, L/min	4.7 ± 1.0	4.0 ± 1.1	**0.014**
Cardiac output post-procedure, L/min	4.1 ± 1.0	4.3 ± 2.0	0.793
**Heart rate, beats per minute**			
Baseline, bpm	71.8 ± 8.6	69.6 ± 15.8	0.688
After first DES, bpm	74.3 ± 13.4	71.4 ± 16.7	0.459
Post-procedure, bpm	76.2 ± 18.3	74.5 ± 16.7	0.696

Abbreviations: DES: Drug eluting stent; HRPCI: high-risk percutaneous coronary intervention; LVEDP: left ventricular end-diastolic pressure; mPAP: mean pulmonary artery pressure; pHRPCI: protected high-risk percutaneous coronary intervention; PCWP, pulmonary capillary wedge pressure. Bold *p*-values indicate statistical significance.

**Table 3:** Laboratory measurements.

Variable, Mean ± Stdv	pHRPCI (*n* = 31)	Non-pHRPCI (*n* = 31)	*p*-Value
**NT-proBNP, pg/mL**			
0 h	4193 ± 4549	5511 ± 6981	0.383
6 h	4781 ± 5929	6578 ± 8419	0.874
12 h	5674 ± 6388	8779 ± 14,389	0.843
24 h	6245 ± 7132	7276 ± 9090	0.633
Troponin *, pg/mL			
0 h	100.6 ± 299.3	51.2 ± 48.3	0.372
6 h	164.8 ± 365	78.7 ± 53.2	0.927
12 h	178.9 ± 307.4	124.8 ± 77.6	0.5
24 h	354.4 ± 610.6	188.6 ± 139.3	0.575
**Creatinine kinase isoenzyme–MB, U/L**			
0 h	20.1 ± 33.9	15.2 ± 5.0	0.432
6 h	26.1 ± 33.3	16.2 ± 6.1	0.134
12 h	33.7 ± 40.8	21.0 ± 9.7	0.314
24 h	43.1 ± 79.9	22.5 ± 10.1	0.502
**Creatinine, mg/dL**			
0 h	1.1 ± 0.4	1.0 ± 0.4	0.271
6 h	1.3 ± 0.4	1.1 ± 0.3	0.185
12 h	1.3 ± 0.4	1.1 ± 0.3	0.135
24 h	1.5 ±0.5	1.2 ± 0.4	0.043
**eGFR, mL/min/1.73 m^2^**			
0 h	70.1 ± 22.2	76.3 ± 22.5	0.284
6 h	63.3 ± 21.7	71.3 ± 21.5	0.159
12 h	61.5 ± 21.0	70.8 ± 21.4	0.097
24 h	53.5 ± 19.8	65.9 ± 21.5	0.022

* highly sensitive. Abbreviations: eGFR: estimated glomerular filtration rate; HRPCI: high-risk percutaneous coronary intervention; NT-proBNP: N-terminal pro brain natriuretic peptide; pHRPCI: protected high-risk percutaneous coronary intervention.

## Data Availability

The data that support the findings of this study are available upon reasonable request from the corresponding author, J.L.

## Abbreviations

AMI	Acute myocardial infarction
CAD	Coronary artery disease
CK	Creatinine kinase
CK–MB	Creatinine kinase isoenzyme–MB
CO	Cardiac output
eGFR	Estimated glomerular filtration rate
HRPCI	High-risk percutaneous coronary intervention
Hs-cTnT	High-sensitive cardiac troponin T
LM	Left main
LVEDP	Left ventricular end-diastolic pressure
LVEF	Left ventricular ejection fraction
MACCE	Major adverse cardiac and cerebrovascular events
MAP	Mean arterial pressure
mPAP	Mean pulmonary artery pressure
NT-proBNP	N-terminal pro brain natriuretic peptide
SD	Standard deviation
PCI	Percutaneous coronary intervention
PCWP	Pulmonary capillary wedge pressure
pHRPCI	Protected high-risk percutaneous coronary intervention
pMCS	Percutaneous mechanical circulatory support
